# Supplementary material for: Endemism patterns are scale dependent
Source: Nat Commun. 2020 Apr 30;11:2115. doi: 10.1038/s41467-020-15921-6 (PMC7192928; doi:10.1038/s41467-020-15921-6)
Supplement: Supplementary file 1 — Supplementary information [file 41467_2020_15921_MOESM1_ESM.pdf]

Supplementary Information

Endemism patterns are scale dependent

Daru et al.

*Corresponding author. Email: barnabas.daru@tamucc.edu

Contents:

Supplementary Figures 1 to 4

Supplementary Tables 1 to 4

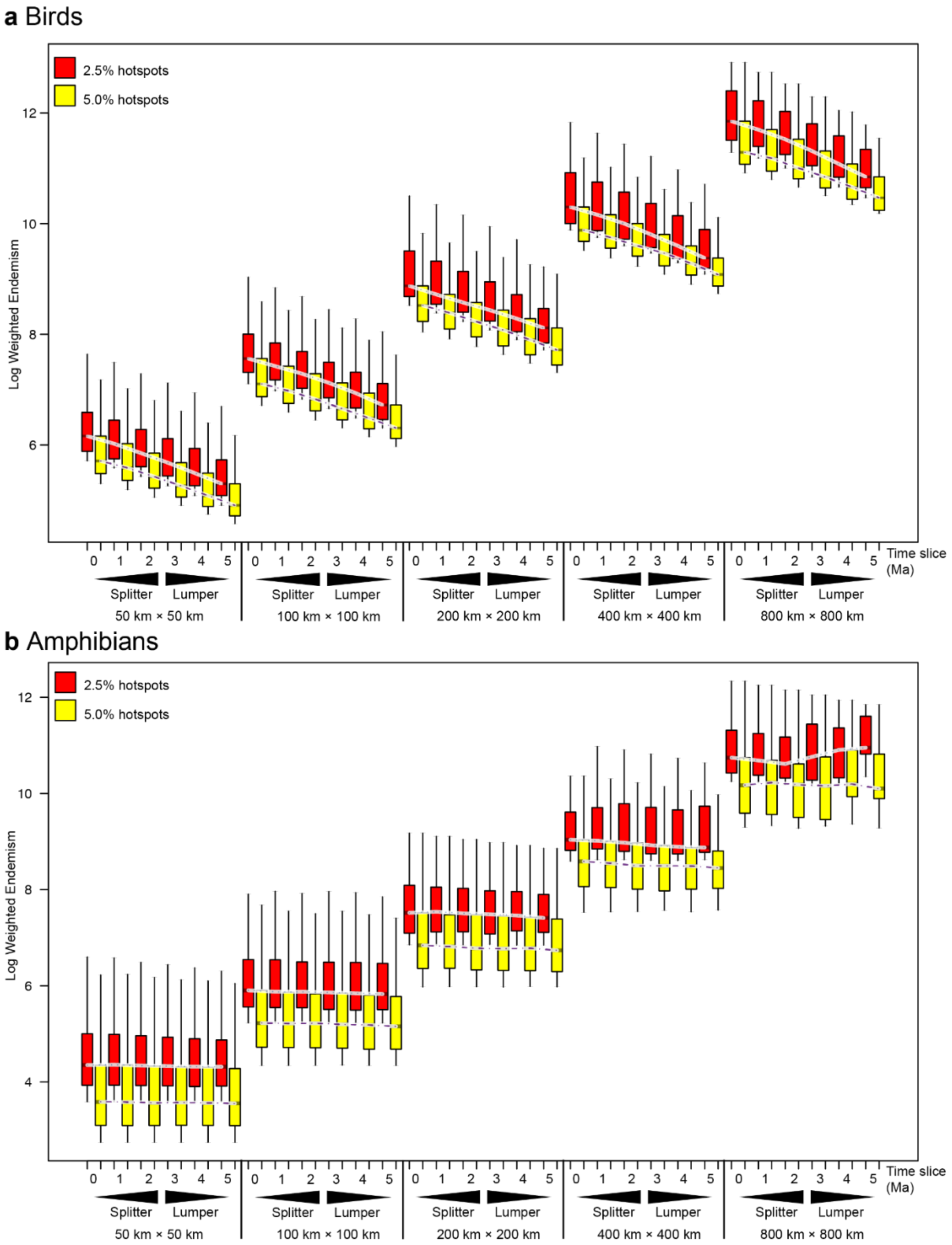

Supplementary Figure 1 | Changes in weighted endemism in relation to grain size (50 × 50, 100 × 100, 200 × 200, 400 × 400, and 800km × 800 km). **a** birds (n = 10,018 species), and **b** amphibians (n = 5872 species). The effect of spatial grain is evident in the fitted slopes between

species endemism and taxonomic treatment (splitting vs lumping across varying time slices). Box plots represent hotspot cells with the highest weighted endemism values (top 2.5% endemism in red and 5.0% endemism in yellow). Lines within the boxes represent the 50th percentile (median), and whiskers represent 2.5^{th} and 97.5^{th} percentiles. Analysis was based on phylogenetic data derived from a random draw of 100 trees from a Bayesian posterior distribution of 600 trees for birds and 10,000 trees for amphibians. We integrated our results across variations of tree topologies and branch lengths for both birds and amphibians by calculating WE for each 100 trees from the posterior distribution and computing the median across grid cells.

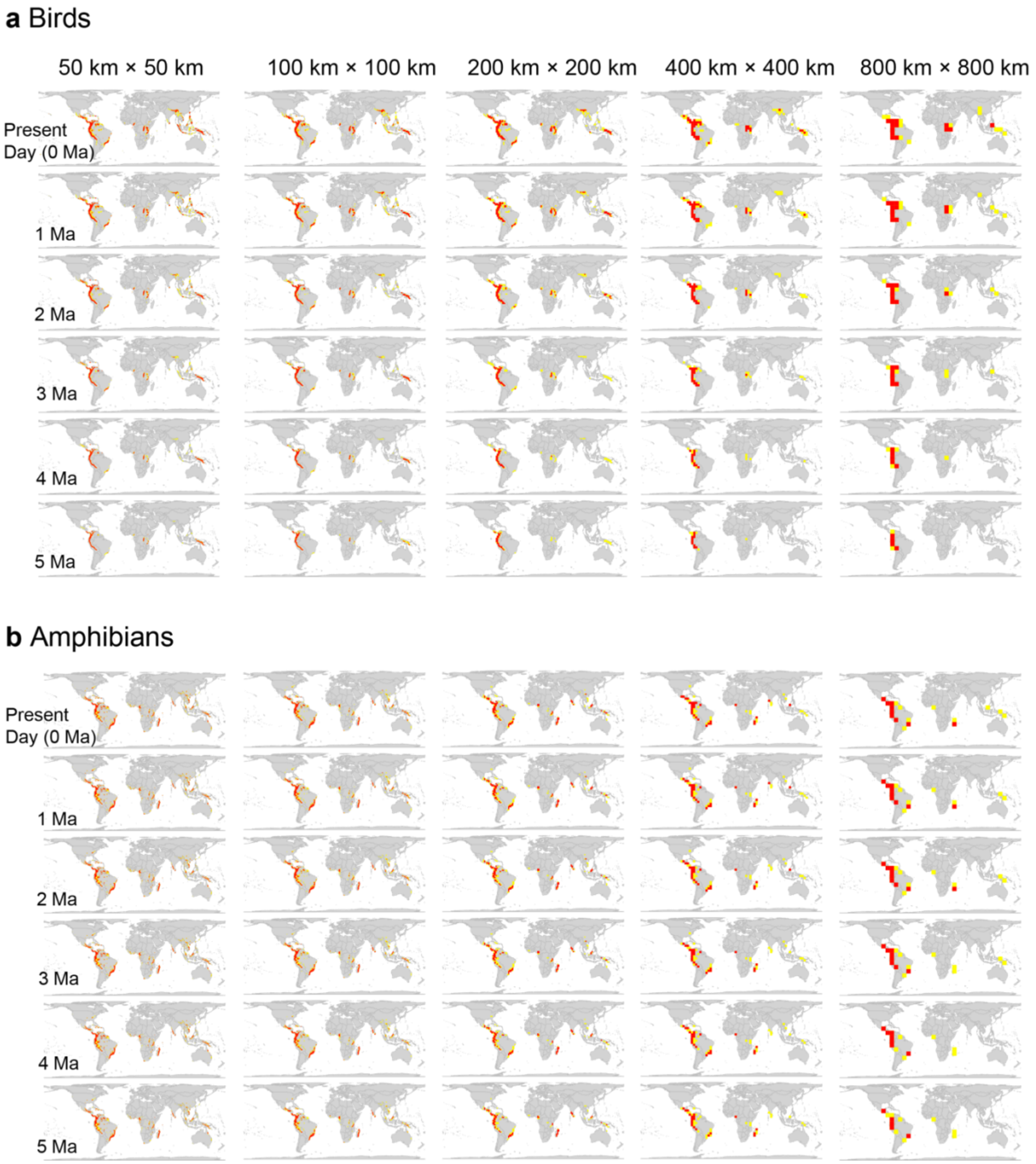

Supplementary Figure 2 | Scale dependence of spatial grain and taxonomic treatment of hotspots of weighted endemism. a birds (n = 10,018 species), and **b** amphibians (n = 5872 species) across grains and taxonomic treatment. Hotspots are defined as the grid cells with the highest 2.5% of weighted endemism (indicated in red), and highest 5% (indicated in yellow). Variations of taxonomic treatments in presented results are based on species' divergence times at varying time depths: present-day, 1, 2, 3, 4, and 5 million years ago (Ma) – replicating increasing 'lumping' of taxa. Analysis of clade collapse based on a randomly selected subset of 100 trees from a posterior distribution of 600 trees for birds and 100 trees from a posterior distribution of 10,000 trees for amphibians. The maps are in Behrmann projection.

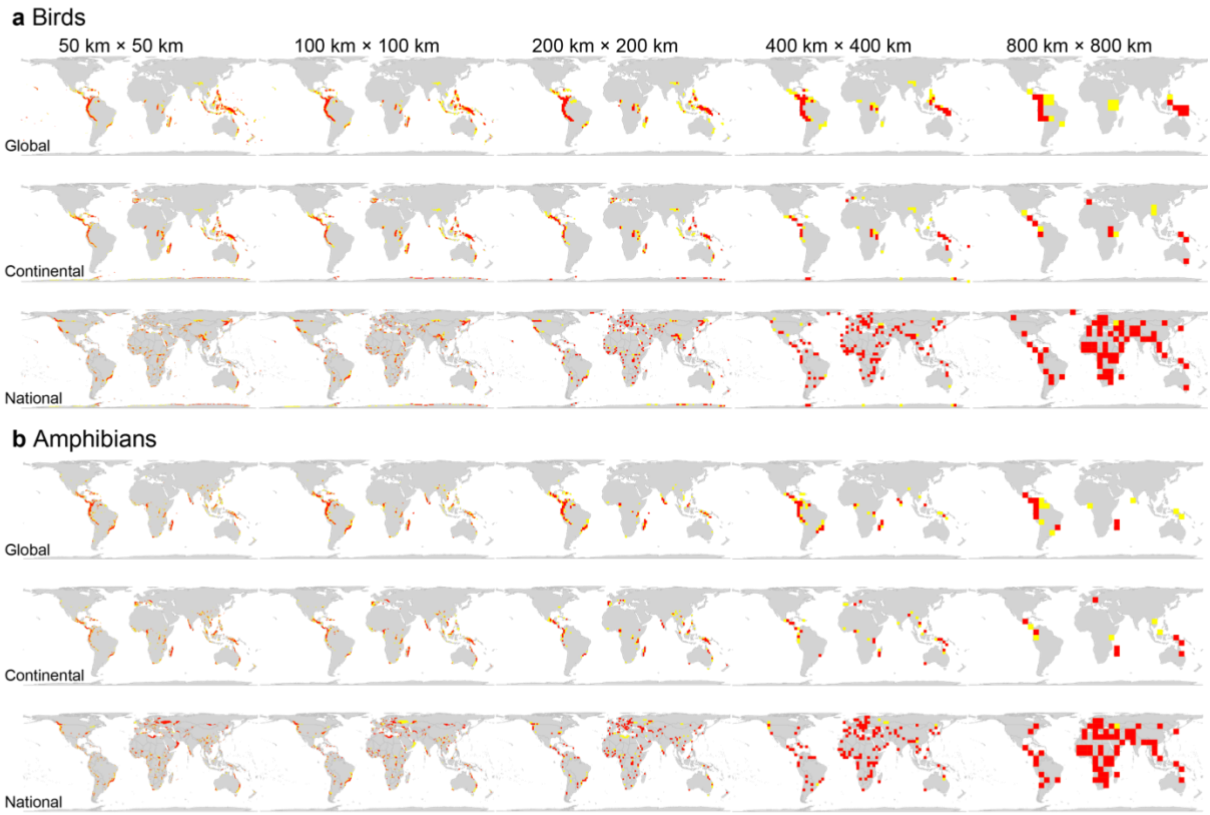

Supplementary Figure 3 | Hotspots of phylogenetic endemism are influenced strongly by spatial extent, varying along global, continental and local extents at country level. a birds (n = 10,018 species), and **b** amphibians (n = 5872 species) of the world across three levels of spatial extents (global, continental, and national) and grain sizes at 50, 100, 200, 400, and 800 km. Hotspots are defined as the grid cells with the highest 2.5% of phylogenetic endemism (indicated in red), and highest 5% (indicated in yellow). The maps are in Behrmann projection.

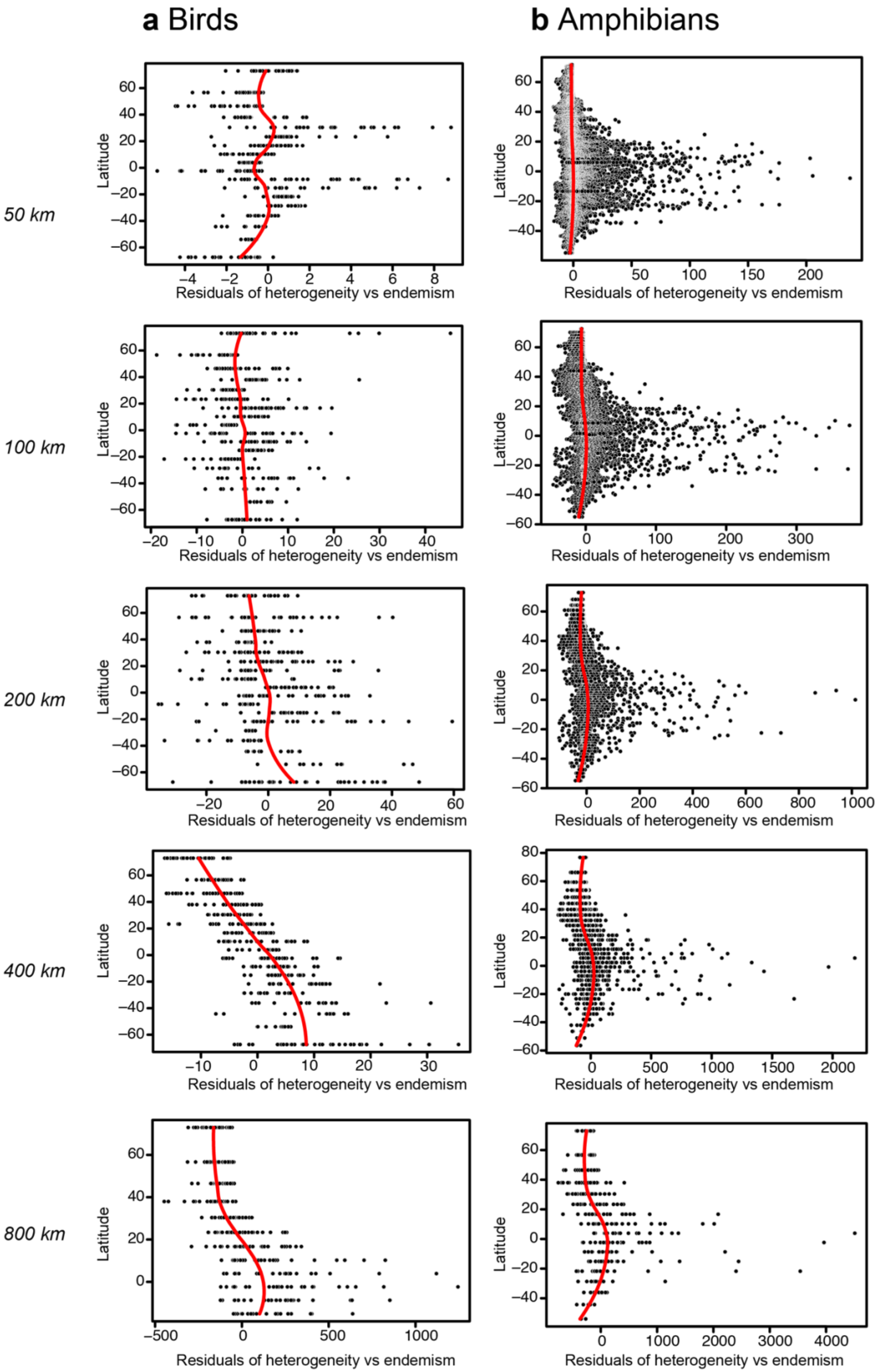
a Birds
b Amphibians
50 km
100 km
200 km
400 km
800 km
Latitude
Residuals of heterogeneity vs endemism

Supplementary Figure 4 | Scatterplot of latitudinal variation with residuals from landscape heterogeneity and phylogenetic endemism. Landscape heterogeneity was measured as standard deviation of altitude per grid cell. Trend line (in red) computed by evaluating the loess smooth at equally spaced points covering the range of endemism values for each latitude.

Supplementary Table 1 | Variation of environmental heterogeneity on patterns of weighted endemism across grain sizes for birds (n = 10,018 species). The variations of environmental variables on weighted endemism are presented as estimated coefficients of the fixed effects and their 95% confidence intervals. Statistics based on a single linear mixed-effects model of endemism with all predictors (temperature variation, precipitation variation, productivity variation, elevation variation, spatial autocovariate and random effects of continent identities for each grid cell) across grain sizes.

Standard deviation of environmental variable	Lower confidence limit	Estimates of fixed effects	Upper confidence limit	P value	Grain size
Altitude	-0.0697578	-0.0524354	-0.035113	3.00E-09	050km
Precipitation	0.10613808	0.11167313	0.11720819	0	050km
Temperature	0.11030747	0.12750172	0.14469597	0	050km
Net Primary Productivity	0.03019083	0.0347439	0.03929697	0	050km
Altitude	-0.0786011	-0.043682	-0.0087629	0.01423973	100km
Precipitation	0.12075586	0.13275049	0.14474512	0	100km
Temperature	0.11402142	0.14856795	0.18311447	0	100km
Net Primary Productivity	0.041402	0.05158408	0.06176615	0	100km
Altitude	-0.1101209	-0.0423614	0.02539799	0.22079209	200km
Precipitation	0.13566482	0.16236575	0.18906668	0	200km
Temperature	0.11641927	0.18303977	0.24966027	7.88E-08	200km
Net Primary Productivity	0.03631477	0.06018126	0.08404775	8.22E-07	200km
Altitude	-0.1294695	-0.0159557	0.09755817	0.78345038	400km
Precipitation	0.0961984	0.15117991	0.20616141	9.80E-08	400km
Temperature	0.09449801	0.20531252	0.31612702	0.00030807	400km
Net Primary Productivity	0.07380615	0.12583492	0.17786369	2.63E-06	400km
Altitude	-0.2192553	-0.0330971	0.15306111	0.73004606	800km
Precipitation	0.18514006	0.31659781	0.44805556	5.28E-06	800km
Temperature	0.00318641	0.1855592	0.36793198	0.04936105	800km
Net Primary Productivity	-0.0200282	0.10062152	0.22127121	0.10661351	800km

Supplementary Table 2 | Variation of environmental heterogeneity on patterns of phylogenetic endemism across grain sizes for birds (n = 10,018 species). The variations of environmental variables on phylogenetic endemism are presented as estimated coefficients of the fixed effects and their 95% confidence intervals. Statistics based on a single linear mixed-effects model of endemism with all predictors (temperature variation, precipitation variation, productivity variation, elevation variation, spatial autocovariate and random effects of continent identities for each grid cell) across grain sizes.

Standard deviation of environmental variable	Lower confidence limit	Estimates of fixed effects	Upper confidence limit	P value	Grain size
Altitude	-0.0409809	-0.0269563	-0.0129317	0.00016535	050km
Precipitation	0.09950663	0.10396322	0.10841981	0	050km
Temperature	0.07919734	0.09310256	0.10700779	0	050km
Net Primary Productivity	0.09542031	0.09919593	0.10297154	0	050km
Altitude	-0.0207115	0.00668556	0.03408259	0.63250473	100km
Precipitation	0.11896088	0.12826214	0.13756341	0	100km
Temperature	0.05065487	0.0777353	0.10481574	1.89E-08	100km
Net Primary Productivity	0.13885163	0.14716632	0.15548102	0	100km
Altitude	-0.030949	0.01903642	0.06902182	0.45570339	200km
Precipitation	0.15064416	0.17011201	0.18957986	0	200km
Temperature	0.03426137	0.0833785	0.13249563	0.00089281	200km
Net Primary Productivity	0.16645224	0.1848674	0.20328256	0	200km
Altitude	-0.0549291	0.02441347	0.10375602	0.54746909	400km
Precipitation	0.11053265	0.1484237	0.18631475	5.13E-14	400km
Temperature	0.02255027	0.1004463	0.17834233	0.01185367	400km
Net Primary Productivity	0.25169128	0.28939436	0.32709744	0	400km
Altitude	-0.0661123	0.05642616	0.17896461	0.37188173	800km
Precipitation	0.21958122	0.30122979	0.38287835	1.43E-11	800km
Temperature	-0.0844244	0.03721912	0.15886262	0.55273609	800km
Net Primary Productivity	0.20271524	0.28045113	0.35818702	3.58E-11	800km

Supplementary Table 3 | Variation of environmental heterogeneity on patterns of weighted endemism across grain sizes for amphibians (n = 5872 species). The variations of environmental variables on weighted endemism are presented as estimated coefficients of the fixed effects and their 95% confidence intervals. Statistics based on a single linear mixed-effects model of endemism with all predictors (temperature variation, precipitation variation, productivity variation, elevation variation, spatial autocovariate and random effects of continent identities for each grid cell) across grain sizes.

Standard deviation of environmental variable	Lower confidence limit	Estimates of fixed effects	Upper confidence limit	P value	Grain size
Altitude	0.00026958	0.02952163	0.05877368	0.04794087	050km
Precipitation	0.05546624	0.06418358	0.07290092	0	050km
Temperature	-0.0276305	0.00135811	0.03034667	0.92684145	050km
Net Primary Productivity	-0.0159709	-0.0086321	-0.0012934	0.02115474	050km
Altitude	-0.0372893	0.01628116	0.0698516	0.55146834	100km
Precipitation	0.08234983	0.09988831	0.11742678	0	100km
Temperature	-0.0207159	0.03222212	0.08516009	0.23297414	100km
Net Primary Productivity	-0.0239149	-0.0091093	0.00569624	0.22795687	100km
Altitude	-0.2117516	-0.1077898	-0.0038281	0.04235557	200km
Precipitation	0.14561445	0.18516426	0.22471408	0	200km
Temperature	0.07196786	0.17416116	0.27635446	0.00085378	200km
Net Primary Productivity	-0.0340667	0.00090057	0.03586784	0.95976928	200km
Altitude	-0.2495879	-0.0753522	0.09888354	0.39799092	400km
Precipitation	0.09143934	0.17245415	0.25346897	3.50E-05	400km
Temperature	0.03324691	0.2019119	0.37057689	0.01949136	400km
Net Primary Productivity	-0.0282016	0.04696284	0.12212725	0.22216597	400km
Altitude	-0.3466574	-0.0892545	0.16814847	0.50137389	800km
Precipitation	0.30778832	0.48210064	0.65641297	2.27E-07	800km
Temperature	-0.0868551	0.16405279	0.4149607	0.20558098	800km
Net Primary Productivity	-0.2907511	-0.131317	0.02811716	0.11125765	800km

Supplementary Table 4 | Variation of environmental heterogeneity on patterns of phylogenetic endemism across grain sizes for amphibians (n = 5872 species). The variations of environmental variables on phylogenetic endemism are presented as estimated coefficients of the fixed effects and their 95% confidence intervals. Statistics based on a single linear mixed-effects model of endemism with all predictors (temperature variation, precipitation variation, productivity variation, elevation variation, spatial autocovariate and random effects of continent identities for each grid cell) across grain sizes.

Standard deviation of environmental variable	Lower confidence limit	Estimates of fixed effects	Upper confidence limit	P value	Grain size
Altitude	-0.0610311	-0.0334587	-0.0058863	0.01739738	050km
Precipitation	0.08170521	0.09030645	0.09890769	0	050km
Temperature	0.05868108	0.08601527	0.11334946	7.00E-10	050km
Net Primary Productivity	0.01268093	0.01968456	0.02668819	3.64E-08	050km
Altitude	-0.0735798	-0.0239604	0.02565897	0.34402295	100km
Precipitation	0.14403282	0.16075672	0.17748062	0	100km
Temperature	0.0392308	0.08825347	0.13727613	0.00042056	100km
Net Primary Productivity	0.0269603	0.04094013	0.05491997	9.78E-09	100km
Altitude	-0.1865975	-0.1013671	-0.0161367	0.01989363	200km
Precipitation	0.21500206	0.2480116	0.28102114	0	200km
Temperature	0.09858867	0.18234554	0.26610241	2.07E-05	200km
Net Primary Productivity	0.03881319	0.06825446	0.09769574	5.82E-06	200km
Altitude	-0.1831175	-0.0429577	0.09720207	0.54912697	400km
Precipitation	0.13980627	0.20444268	0.26907908	1.01E-09	400km
Temperature	0.02380214	0.16082139	0.29784065	0.02199318	400km
Net Primary Productivity	0.10984347	0.17242758	0.2350117	9.51E-08	400km
Altitude	-0.2529514	-0.0420803	0.16879094	0.69869629	800km
Precipitation	0.19600254	0.33721602	0.4784295	6.54E-06	800km
Temperature	-0.0914024	0.11691133	0.32522501	0.27697901	800km
Net Primary Productivity	0.02910393	0.16264744	0.29619095	0.01899494	800km
