# Supplementary material for: Endemism patterns are scale dependent
Source: Nat Commun. 2020 Apr 30;11:2115. doi: 10.1038/s41467-020-15921-6 (PMC7192928; doi:10.1038/s41467-020-15921-6)
Supplement: Supplementary file 3 — Reporting Summary [file 41467_2020_15921_MOESM3_ESM.pdf]

nature research

Corresponding author(s): BARNABAS H. DARU

Last updated by author(s): Feb 23, 2020

Reporting Summary

Nature Research wishes to improve the reproducibility of the work that we publish. This form provides structure for consistency and transparency in reporting. For further information on Nature Research policies, see Authors & Referees and the Editorial Policy Checklist .

Statistics

For all statistical analyses, confirm that the following items are present in the figure legend, table legend, main text, or Methods section.

n/a	Confirmed	
☐	☒	The exact sample size (*n*) for each experimental group/condition, given as a discrete number and unit of measurement
☐	☒	A statement on whether measurements were taken from distinct samples or whether the same sample was measured repeatedly
☒	☐	The statistical test(s) used AND whether they are one- or two-sided<br>*Only common tests should be described solely by name; describe more complex techniques in the Methods section.*
☐	☒	A description of all covariates tested
☐	☒	A description of any assumptions or corrections, such as tests of normality and adjustment for multiple comparisons
☐	☒	A full description of the statistical parameters including central tendency (e.g. means) or other basic estimates (e.g. regression coefficient) AND variation (e.g. standard deviation) or associated estimates of uncertainty (e.g. confidence intervals)
☐	☒	For null hypothesis testing, the test statistic (e.g. *F*, *t*, *r*) with confidence intervals, effect sizes, degrees of freedom and *P* value noted<br>*Give P values as exact values whenever suitable.*
☒	☐	For Bayesian analysis, information on the choice of priors and Markov chain Monte Carlo settings
☒	☐	For hierarchical and complex designs, identification of the appropriate level for tests and full reporting of outcomes
☒	☐	Estimates of effect sizes (e.g. Cohen's *d*, Pearson's *r*), indicating how they were calculated

Our web collection on statistics for biologists contains articles on many of the points above.

Software and code

Policy information about availability of computer code

Data collection	phyloregion version 0.1.0 (an R package)
Data analysis	All scripts and code necessary to repeat the analyses described here have been made available in the new R package phyloregion (Daru et al. 2020). Also, all data necessary to repeat the analyses described here have been made publicly available through the Dryad digital data repository (https://doi.org/10.5061/dryad.wh70rxwhs).<br><br>Daru, B. H., Karunarathne P., & Schliep K. phyloregion: R package for biogeographic regionalization and spatial conservation. Preprint at https://doi.org/10.1101/2020.02.12.945691 (2020).

For manuscripts utilizing custom algorithms or software that are central to the research but not yet described in published literature, software must be made available to editors/reviewers. We strongly encourage code deposition in a community repository (e.g. GitHub). See the Nature Research guidelines for submitting code & software for further information.

Data

Policy information about availability of data

All manuscripts must include a data availability statement. This statement should provide the following information, where applicable:

- Accession codes, unique identifiers, or web links for publicly available datasets
- A list of figures that have associated raw data
- A description of any restrictions on data availability

The amphibian and bird occurrence range maps included in this study are all publicly available on the IUCN Red List database (https://www.iucnredlist.org/resources/spatial-data-download). The phylogenies used for both birds and amphibians are published phylogenies that are already available in public repositories. Specifically, the bird phylogeny was downloaded from https://birdtree.org (Jetz et al. 2012, Nature), whereas the amphibian phylogeny is also published and publicly available on Dryad at https://doi.org/10.5061/dryad.cc3n6j5

Field-specific reporting

Please select the one below that is the best fit for your research. If you are not sure, read the appropriate sections before making your selection.

[ ] Life sciences [ ] Behavioural & social sciences [x] Ecological, evolutionary & environmental sciences

For a reference copy of the document with all sections, see nature.com/documents/nr-reporting-summary-flat.pdf

Ecological, evolutionary & environmental sciences study design

All studies must disclose on these points even when the disclosure is negative.

Study description	Using breeding bird and amphibian distributions from the IUCN Redlist database in combination with comprehensive published phylogenies, we calculated weighted endemism and phylogenetic endemism separately for all species of birds and amphibians across the globe, by varying the analyses across grain sizes (50, 100, 200, 400 and 800 km), spatial extents (country, continental and global) and taxonomic treatments (taxonomic lumping vs taxonomic splitting). We then tested the effect of environmental heterogeneity using published data on four environmental variables: temperature, altitude, precipitation and productivity. Last, we tested the degree of protection of endemism across scales by the current network of protected areas. Data on protected areas is publicly available at the World Database on Protected Areas (http://protectedplanet.net/).
Research sample	We evaluated breeding bird distributions separately for all birds and amphibians globally using a publicly available dataset of GIS extent-of-occurrence polygons. The dataset for birds are available by request from BirdLife International. 10,018 bird species were included in the analysis. The dataset for amphibians is publicly available for download from the IUCN Red List database (https://www.iucnredlist.org/resources/spatial-data-download). 5872 amphibian species were included in the analysis.
Sampling strategy	No applicable
Data collection	Data was obtained by B.H.D from the IUCN redlist for the range maps, whereas the phylogenies were obtained from Dryad and https://birdtree.org for amphibians and birds, respectively.
Timing and spatial scale	The spatial scale is global. As the aim of the paper is to test the effect of scale on patterns of endemism, we also analyzed the data at various scales along spatial grain (50 km, 100km to 800 km) and spatial extent (country, continental and global).<br>The timing is contemporary times.
Data exclusions	We removed coastal grid cells with < 50% land from analyses to minimize the influence of unequal sampling area.
Reproducibility	We share all scripts and code necessary to repeat the analyses described in this study in a new R package phyloregion (https://github.com/darunabas/phyloregion). In addition, all data necessary to repeat the analyses described here have been made available through the Dryad digital data repository (https://doi.org/10.5061/dryad.wh70rxwhs).
Randomization	We used the function tree_sampler in our phyloregion package to randomly sample a subset of trees from a posterior distribution of phylogenetic trees derived from multiple runs of MrBayes analysis for both amphibians and birds. We then integrated our results across variations of tree topologies and branch lengths for both birds and amphibians by repeating the weighted endemism and phylogenetic endemism calculations for each subset of trees from the posterior distribution of phylogenetic trees and taking the median across grid cells.
Blinding	Blinding was not relevant to the study, as there were no experimental treatments.

Did the study involve field work? [ ] Yes [x] No

Reporting for specific materials, systems and methods

We require information from authors about some types of materials, experimental systems and methods used in many studies. Here, indicate whether each material, system or method listed is relevant to your study. If you are not sure if a list item applies to your research, read the appropriate section before selecting a response.

Materials & experimental systems

n/a	Involved in the study	
[x]	[ ]	Antibodies
[x]	[ ]	Eukaryotic cell lines
[x]	[ ]	Palaeontology
[x]	[ ]	Animals and other organisms
[x]	[ ]	Human research participants
[x]	[ ]	Clinical data

Methods

n/a	Involved in the study	
[x]	[ ]	ChIP-seq
[x]	[ ]	Flow cytometry
[x]	[ ]	MRI-based neuroimaging
